# An Extended Application of the Fast Multi-Locus Ridge Regression Algorithm in Genome-Wide Association Studies of Categorical Phenotypes

**DOI:** 10.3390/plants13172520

**Published:** 2024-09-07

**Authors:** Jin Zhang, Bolin Shen, Ziyang Zhou, Mingzhi Cai, Xinyi Wu, Le Han, Yangjun Wen

**Affiliations:** College of Science, Nanjing Agricultural University, Nanjing 210095, China; zhangjin@njau.edu.cn (J.Z.); 2022111008@stu.njau.edu.cn (B.S.); 2019111004@njau.edu.cn (Z.Z.); 2019111003@njau.edu.cn (M.C.); 2021111004@stu.njau.edu.cn (X.W.); 2022111007@stu.njau.edu.cn (L.H.)

**Keywords:** binary or ordinal traits, multi-locus model, mixed linear model, polygenic background control, ridge regression

## Abstract

Categorical (either binary or ordinal) quantitative traits are widely observed to measure count and resistance in plants. Unlike continuous traits, categorical traits often provide less detailed insights into genetic variation and possess a more complex underlying genetic architecture, which presents additional challenges for their genome-wide association studies. Meanwhile, methods designed for binary or continuous phenotypes are commonly used to inappropriately analyze ordinal traits, which leads to the loss of original phenotype information and the detection power of quantitative trait nucleotides (QTN). To address these issues, fast multi-locus ridge regression (FastRR), which was originally designed for continuous traits, is used to directly analyze binary or ordinal traits in this study. FastRR includes three stages of continuous transformation, variable reduction, and parameter estimation, and it can computationally handle categorical phenotype data instead of link functions introduced or methods inappropriately used. A series of simulation studies demonstrate that, compared with four other continuous or binary or ordinal approaches, including logistic regression, FarmCPU, FaST-LMM, and POLMM, the FastRR method outperforms in the detection of small-effect QTN, accuracy of estimated effect, and computation speed. We applied FastRR to 14 binary or ordinal phenotypes in the *Arabidopsis* real dataset and identified 479 significant loci and 76 known genes, at least seven times as many as detected by other algorithms. These findings underscore the potential of FastRR as a very useful tool for genome-wide association studies and novel gene mining of binary and ordinal traits.

## 1. Introduction

Categorical (either binary or ordinal) quantitative traits are very important and widely observed to measure count and resistance in plants and crop cultivars. For example, the presence (1) or absence (0) of rolled leaves, and susceptibility (1) or resistance (0) to pests are binary phenotypes. The level of leaf serration at 22 °C, ranging from 0 (entire lamina) to 1.5 (sharp/jagged serration) in *Arabidopsis* thaliana, and the infection type, with scores of 0~4, for leaf rust in wheat, are ordinal phenotypic responses. Ordinal traits are represented by an ordered series of numeric value (degree of infections, 0, 2, 3, etc.).

Categorical traits, as special cases of quantitative traits, present a discontinuous distribution of phenotypes, and breeding tests show phenotypic features that cannot be readily explained by simple strict Mendelian inheritance [1]. Many traits with low heritability have ordered categorical scores, such as susceptibility or resistance to a disease, and they exhibit less genetic information [2]. Thus, genetic mechanisms of categorical traits are complex, involving polygenic background control, environment modification, gene expression, gene–environment interaction, and gene–gene interaction [3]. Research on categorical traits is crucial to improving the yield and quality of crops, as well as genetic breeding in plants.

Genome-wide association studies (GWAS), as a hot topic, have generated great interest and provided numerous well-known tools in recent decades. Among them, PLINK is the most widely used tool for GWAS analysis as a standard method [4]. However, PLINK does not account for polygenic backgrounds, which can lead to high false discovery rates, and it can only handle continuous and binary traits. Mixed linear model (MLM), also called linear mixed model (LMM), approaches have been widely used to account for sample relatedness and population structure in GWAS for continuous and binary phenotypes [5,6,7]. Consequently, the number of MLM-based computational tools for genetic dissection is rapidly increasing, including EMMA [8], FaST-LMM [9], GEMMA [10], BOLT-LMM [11], and FastGWA [12], all for continuous traits, and GMMAT [6] and SAIGE [7] via logistic mixed models for binary traits. In addition, Bayes-GLMM [13] has been proposed to implement generalized linear mixed models in a Bayesian framework. And it takes a linear regression model, logistic regression model, and ordered logistic regression model as likelihood functions of continuous, binary, and ordinal traits, respectively, and can analyze continuous and categorical data. Meanwhile, POLMM [5] has been proposed via proportional odds logistic mixed models and it can handle both binary and ordinal traits. However, PLINK and all the above MLM methods are single-locus GWAS algorithms that focus on one marker at a time and require multiple-test correction, and Bonferroni thresholds are overly strict, which may result in the missing of some important small-effect loci associated with traits, thus reducing the detection power [14]. There have been significant efforts in the development of single-locus methods to address this issue. For example, Xu et al. [15] proposed a model-based clustering method for binary traits that utilizes the dependency structure between single-nucleotide polymorphisms (SNPs) by grouping them into three clusters and by pre-specifying the prior distribution patterns of clusters, which better controls the false discovery rate and provides higher power. However, their method cannot handle situations when the direction of the effect changes due to population stratification.

Suitable ways to handle multiple SNPs under population stratification and polygenic background control include multi-locus MLM methodologies that utilize dimension reduction through variable selection, such as FarmCPU [16], mrMLM [17], pLARmEB [18], FASTmrEMMA [19,20], FASTmrMLM [21], and FastRR [22]. All these approaches, while rapid, are designed for continuous phenotypes. Furthermore, for binary traits, both EBLASSO-NE and EBLASSO-NEG [23] were proposed based on a logistic regression model with two different prior distributions in a Bayesian framework. A hierarchical generalized linear mixed model [24] was proposed and applied to ordinal traits in crop cultivars. Nevertheless, the three approaches mentioned for categorical phenotypes could not be applicable to large-scale data due to their low computing efficiency.

Categorical data are often analyzed via generalized linear models (GLM) or generalized linear mixed models (GLMM) by introducing link functions, which increases computational cost. Accordingly, current approaches commonly treat the categorical phenotype as a continuous trait to inappropriately fit the linear regression or mixed linear models, which can cause the biased effect estimates due to the violation of the constant residual variance assumption [5,6,13]. Another approach is to dichotomize the ordinal phenotype into a binary trait with a cutoff, followed by applying a logistic regression or logistic mixed-model method. This approach suffers from phenotypic information loss and, therefore, is less powerful [5,6,13]. In practice, the latter strategy mentioned is commonly used in humans, and rarely in plants. Plants and crop cultivars tend to use the former strategy. For example, for leaf rust in wheat, the infection type is an ordinal response with scores of 0~4. Kaur et al. [25] converted infection type scores to the best linear unbiased estimates as continuous responses and then applied FarmCPU for further analysis. For the presence of rolled leaves or the level of serration in *Arabidopsis* thaliana, Liu et al. [16] treated these binary or ordinal traits as continuous data, and then employed the FarmCPU method. Regardless, as argued previously, neither of these strategies would be appropriate.

Recently, Zhang et al. [22] proposed fast multi-locus ridge regression (FastRR) for continuous phenotypes, which efficiently handles datasets where the number of markers significantly exceeds the sample size—a scenario in which most penalization methods typically struggle. In this paper, we extend FastRR to directly apply to binary and ordinal traits in genome-wide association studies. The algorithm first converts binary or ordinal phenotypes into continuous data by correcting for polygenic background and population structure, rather than using link functions. Then, it screens a small number of potential candidate loci based on correlation to construct a multi-locus model. Finally, it implements parameter estimation using deshrinking ridge regression to identify significant loci associated with the binary or ordinal traits of interest. A series of simulated as well as *Arabidopsis* thaliana real data analyses are used to verify the performance of FastRR in categorical phenotypes. Four other existing continuous/binary/ordinal approaches, including logistic regression [26], FarmCPU [16], FaST-LMM [9], and POLMM [5], are used for comparison analysis. Collectively, this work provides the implementation of an alternative GWAS approach for binary and ordinal phenotypes and ultimately contributes toward identifying the genetic mechanisms of complex traits in plants and crop cultivars.

## 2. Materials and Methods

### 2.1. The Calculation of the Mixed Linear Model

Let $y_{i}(i=1,2,\ldots ,n)$ be the binary or ordinal phenotype value of the ith individual in a sample of size n from a natural population, and the MLM can be described as follows:

(1) y=Wα+Gβ+u+ε

where $y={\left(y_{1},y_{2},\cdots ,y_{n}\right)}^{T}$ is an n×1 vector of phenotype value. α is a c×1 fixed effect vector, including the population structure, principal component, intercept, and so on; W is the correspondingly designed matrix for α, whose dimension is n×c; G is an n×1 vector of marker genotypes, $\beta \sim N(0,\sigma_{\beta }^{2})$ is a random effect of putative QTN, and $\sigma_{\beta }^{2}$ is variance of putative QTN; $u\sim MVN(0,\sigma_{u}^{2}K)$ is an n×1 vector of the polygenic effect, K is an n×n known kinship matrix, and $\sigma_{u}^{2}$ is the variance of polygenic background; $\varepsilon \sim MVN(0,\sigma^{2}I_{n})$ is an n×1 vector of the residual, $I_{n}$ is an n×n identity matrix, and $\sigma^{2}$ is residual variance. N and MVN denote a univariate and multivariate normal distribution, respectively.

As β is treated as a random effect, the variance of y in model (1) is as follows:

(2) $$Var\left(y\right)=\sigma_{\beta }^{2}GG^{T}+\sigma_{u}^{2}K+\sigma^{2}I_{n}=\sigma^{2}\left(\lambda_{\beta }GG^{T}+\lambda_{u}K+I_{n}\right)$$

where

$$\lambda_{\beta }=\sigma_{\beta }^{2}/\sigma^{2},\;\lambda_{u}=\sigma_{u}^{2}/\sigma^{2}$$

### 2.2. Fast Multi-Locus Ridge Regression Algorithm

**Figure 1:**
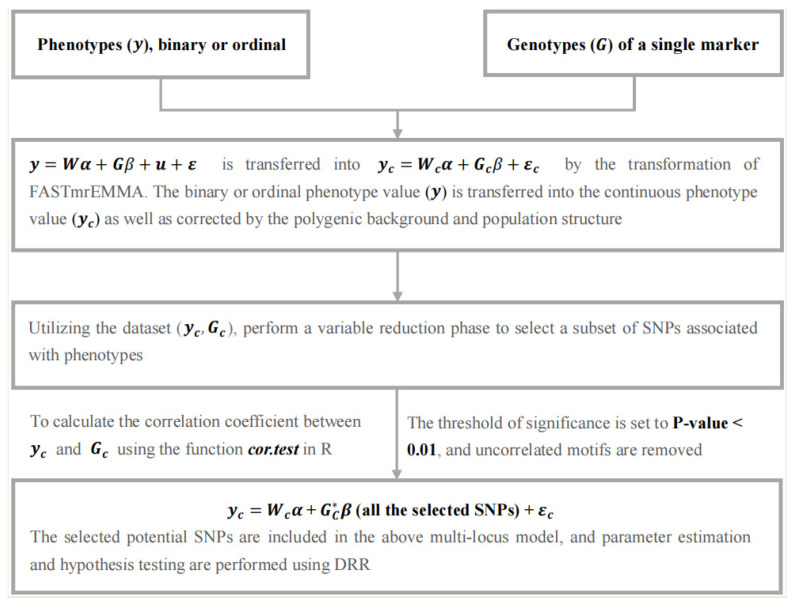
A flow chart of the FastRR method.

The FastRR algorithm is a multi-stage flexible approach for GWAS, which simultaneously implements detection and estimation for associated loci. We describe it with the following stages (Figure 1).

#### 2.2.1. Continuous-Transformed Stage

A transformation matrix is generated using the FASTmrEMMA method [19,20] in this stage. The key point of solving the model (1) is to estimate two ratios of variance components, $\lambda_{\beta }$ and $\lambda_{u}$, which cause expensive computational burden. It is noted that polygenic variance is always larger than zero, while variance of the majority of SNPs is zero, because these loci are not associated with the trait, which means $\lambda_{\beta }=0$. Therefore, in model (1), we delete Gβ, and estimate ${\hat{\lambda }}_{u}$ with the reduced model with only the polygenic background, and replace $\lambda_{u}$ by ${\hat{\lambda }}_{u}$ in model (3) [19,20], avoiding the re-estimation of $\lambda_{u}$ for each single-marker scan, thus

(3) $$Var\left(y\right)=\sigma^{2}\left(\lambda_{\beta }GG^{T}+{\hat{\lambda }}_{u}K+I_{n}\right)=\sigma^{2}\left(\lambda_{\beta }GG^{T}+B\right)$$

where

$$B={\hat{\lambda }}_{u}K+I_{n}$$

An eigen decomposition of the positive definite matrix B is:

(4) $$B=Q\Lambda Q^{T}=\left(Q\Lambda^{\frac{1}{2}}Q^{T}\right)\left(Q\Lambda^{\frac{1}{2}}Q^{T}\right)$$

in which Q is orthogonal and Λ is a diagonal matrix with positive eigenvalues. Let $C=Q\Lambda^{-\frac{1}{2}}Q^{T}$; the model (1) is changed to:

(5) $$y_{C}=W_{C}\alpha +G_{C}\beta +\varepsilon_{C}\;\;\;\;\;\;\;\;\;$$

where

$$y_{C}=Cy,W_{C}=CW,G_{C}=CG,\varepsilon_{C}=Cu+C\varepsilon ,\varepsilon_{C}\sim MVN(0,\sigma^{2}I_{n})$$

Through this step in model (5), we transform binary or ordinal into continuous phenotype values for subsequent analysis. At the same time, FastRR also fully considers polygenic background and population structure.

#### 2.2.2. Variable Reduction Stage

Numerous studies have illustrated that most quantitative traits are controlled by a small part of genes, including a few genes with large effects and poly genes with small effects [18,27]. Thus, it is important to dissect all significantly associated loci from a large number of markers. Here, we perform a variable reduction phase in FastRR to detect a subset of variables associated with phenotypes, with the aim of reducing the computational complexity of high-dimensional analysis.

We calculate the correlation coefficient between $y_{C}$ and $G_{C}$ in model (5) for each marker, and the function cor.test in R returns the *p*-value of the correlation test. The threshold of significance was set to a *p*-value < 0.01 [28] and uncorrelated motifs were removed. At the next stage, all potential loci are selected to construct a reduced multi-locus model. Essentially, this correlation step is similar to the single-marker scanning, which combines with the polygenic background without considering the variance component $\sigma_{\beta }^{2}$.

#### 2.2.3. Parameter Estimation Stage

In the reduced multi-locus model,

(6) $$y_{C}=W_{C}\alpha +G_{C}^{*}\beta +\varepsilon_{C}$$

where $y_{C}$ is the continuous-transformed phenotype vector of quantitative traits, α is the vector of fixed effects, $W_{C}$ is the corresponding design matrix for α, $\varepsilon_{C}\sim MVN(0,\sigma^{2}I_{n})$, and$\;\sigma^{2}$ is residual variance, all of which are the same as in model (5). $\beta ={\left(\beta_{1,}\beta_{2},\ldots ,\beta_{q}\right)}^{T}$ is a q×1 random effect vector of the selected q SNPs from the above step, and $\beta_{k}\sim N\left(0,\varphi^{2}\right),k=1,2,\cdots ,q$, $\varphi^{2}$ is variance of potential associated markers; $G_{C}^{*}$ is an n×q genotype matrix of q markers after continuous transformation. Here, the polygenic background is not considered in model (6), because in the above two steps we have selected all potential associated QTNs under polygenic background. All parameters in model (6) are estimated by deshrinking ridge regression (DRR) [29].

The estimated effect and its variance for the DRR for the kth marker are

(7) $${\hat{\beta }}_{k}^{DRR}{=({{G_{C\;k}^{*}}^{T}H}^{-1}G_{C\;k}^{*})}^{-1}{G_{C\;k}^{*}}^{T}H^{-1}\left(y_{C}-W_{C}\alpha \right)\;\;\;$$

(8) $$\mathrm{var}({\hat{\beta }}_{k}^{DRR})={\left({G_{C\;k}^{*}}^{T}H^{-1}G_{C\;k}^{*}\right)}^{-1}\sigma^{2}-\varphi^{2}\;\;\;\;,$$

respectively, where $H=\frac{\varphi^{2}}{\sigma^{2}}\left(G_{C}^{*}{G_{C}^{*}}^{T}\right)+I_{n},G_{C\;k}^{*}$ is the kth column of matrix $G_{C}^{*}$. Therefore, the Wald test statistic of DRR is

(9) $$W_{k}=\frac{({\hat{\beta }}_{k}^{DRR}{)}^{2}}{\mathrm{var}({\hat{\beta }}_{k}^{DRR})}\;\;$$

which follows a Chi-square distribution with one degree of freedom under the null model, $H_{0}:\beta_{k}=0$. Bonferroni correction was used and the significance threshold was set to 0.05/q in the analysis [29].

### 2.3. Comparison Methods

#### 2.3.1. Logistic Regression

A generalized linear model (GLM) [26] is a generalization of the general linear model, which can be applied to continuous, binary, and count data. R software (Version 4.2.1) provides the function *glm*() for fitting generalized linear models. When the parameter ‘family’ is set to ‘binomial’, it specifies a logistic regression model for a binary trait, which is equivalent to using ‘--logistic’ in PLINK. Currently, the function *glm*() has not been designed for a multinomial distribution family, so we dichotomized the ordinal trait to a binary phenotype for input by defining 1 or 0 depending on whether the ordinal value is more than its mean. Given the issues of computational costs, we constructed a single-locus model and performed logistic regression for each marker. The function *glm*() can be found at https://search.r-project.org/CRAN/refmans/rms/html/Glm.html (accessed on 15 May 2023).

#### 2.3.2. FarmCPU

FarmCPU, which is a multi-locus MLM method, was proposed by Liu et al. [16]. To completely eliminate the confounding between testing markers and kinship, FarmCPU divides a multi-locus mixed model into two parts: a fixed-effect model (FEM) and a random effect model (REM), and uses them iteratively. An FEM features testing markers, one at a time, and multiple associated markers as covariates to control false positives. These associated markers are named as pseudo QTNs. To avoid model over-fitting problems in FEMs, pseudo QTNs are estimated by an REM, where the pseudo QTNs are used to define kinship. An FEM and REM are used iteratively until no change occurs on the pseudo QTNs. FarmCPU is designed for continuous data, so we employed it by treating the binary or ordinal trait as a continuous phenotype for input. The method was implemented by the R package GAPIT (https://www.zzlab.net/GAPIT/ (accessed on 5 June 2023)).

#### 2.3.3. FaST-LMM

The linear mixed model (LMM) tackles confounders by using measures of genetic similarity to capture the probabilities that pairs of individuals have causative alleles in common. For large-scale datasets, the time required to construct a genetic similarity matrix using all SNPs is too long, and the memory required is too large. To address this issue, FaST-LMM [9], which is a single-locus model, builds a realized relationship matrix by partially sampling 200~2000 markers, which improves computational efficiency. However, this algorithm is used to analyze continuous quantitative traits, not for binary or ordinal traits. Therefore, we also employed FaST-LMM by treating the binary or ordinal trait as a continuous phenotype for input. The method was implemented by the R package GAPIT (https://www.zzlab.net/GAPIT/( accessed on 12 June 2023)).

#### 2.3.4. POLMM

POLMM [5] is a recently designed single-locus GWAS method for the ordinal trait using a proportional odds logistic mixed model. POLMM performs penalized quasi-likelihood and average information restricted maximum likelihood algorithms to efficiently fit the mixed model, and uses saddle-point approximation to calculate the *p*-value. It can effectively control the type I error rate. The algorithm was implemented by the POLMM software package (Version 0.2.3) (https://github.com/WenjianBI/POLMM (accessed on 3 July 2023)).

In summary, among the above four comparison methods, logistic regression is used for binary traits, FarmCPU and Fast-LMM are designed for continuous traits, POLMM can handle binary or ordinal traits. The objective of this study is to directly apply the FastRR algorithm to binary and ordinal traits and to evaluate its performance in the QTN detection of categorical phenotypes. Bonferroni correction was used in all comparison methods.

### 2.4. Experimental Materials

#### 2.4.1. Simulation Data

We generated genotypes according to the minor allele frequency (MAF) in the interval (0.1, 0.5) under Hardy–Weinberg equilibrium. The simulation datasets contained 2000 individuals with 10,000 genetic variants. The total average and residual variance were both set at 10.0. On this basis, three simulation experiments were generated from the following mixed linear models.

For the first simulation experiment, a fixed-position QTN was simulated placed on SNP 98 with a small effect size of 0.461. For the second simulation experiment, five fixed-position QTNs were placed on SNP 98, 301, 540, 801, and 1000, with effects of 0.545, 0.862, 0.860, 1.079, and 1.209, respectively. For the third simulation experiment, we randomly selected 10 QTNs with an MAF > 0.3, and the total heritability of 10 QTNs was less than 19%, among which the maximum was 3.16% and the minimum was 0.523%. Additionally, three scenarios for each simulation experiment were considered in a mixed linear model due to the varying degree of polygenic backgrounds among different species, including two times the polygenic background (2 k), five times the polygenic background (5 k), and ten times the polygenic background (10 k).

To investigate the performance of different distribution and hierarchical levels for each of the above 9 combinations, we also considered the representative combinations between three types of phenotypic distribution and hierarchical level number: a normal distribution with five hierarchical levels ranging from 1 to 5 (Figure S1A,D,G), a uniform distribution with five hierarchical levels ranging from 1 to 5 (Figure S1B,E,H), and a binomial distribution with two hierarchical levels, that is, binary phenotype data with values of 0 and 1 (Figure S1C,F,I). Each of the total 27 simulation scenarios was repeated 100 times. All analyses were conducted on 96 CPU cores of Intel Xeon Platinum 8168 M processor at 2.70 GHz and 314 GB RAM.

The statistical power for each QTN detected (power) was defined as the proportion of samples over the Bonferroni threshold to the total number for the 100 replications. The average estimated effect (mean) was defined as the mean value of the effect estimated for 100 replications of the QTN for a fixed location. MSE represented the mean squared error, which is the average of the sum of squares of the differences between the estimated effect and the true value, and can be used to evaluate the accuracy of effect estimates. A smaller MSE value indicates a more accurate estimation of the algorithm, and, conversely, a larger MSE value indicates a lower accuracy. The Receiver Operating Characteristic (ROC) curve shows the statistical power under different Type I errors, which is an important index for evaluating the performance of a model. The heritability for each locus (*r*^2^) was defined as the ratio of genotypic variance for each QTN to phenotypic variance.

#### 2.4.2. Arabidopsis Data

**Figure 2:**
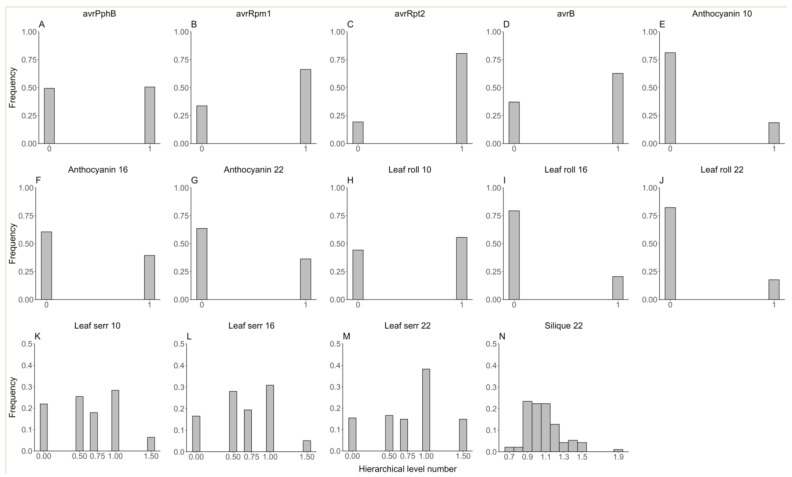
The phenotypic distribution of fourteen binary or ordinal traits in the *Arabidopsis* real dataset. (**A**–**J**) for ten binary traits (avrPphB, avrRpm1, avrRpt2, avrB, Anthocyanin 10, 16, and 22, Leaf roll 10, 16, and 22); (**K**–**N**) for four ordinal traits (Leaf serr 10, 16, 22, and Silique 22).

To evaluate the performance of FastRR for categorical phenotypes, we reanalyzed the genetic datasets of *Arabidopsis* published by Atwell et al. [30]. A total of 199 *Arabidopsis* lines and 216,130 SNPs were obtained from https://www.arabidopsis.org/ (accessed on 16 May 2024). Among 107 traits, we analyzed 14 binary or ordinal traits (Figure 2), including 10 binary traits: avrPphB (presence or absence of avrPphB), avrRpm1 (presence or absence of avrRpm1), avrRpt2 (presence or absence of avrRpt2), avrB (presence or absence of avrB), Anthocyanin 10, 16, and 22 (presence or absence of anthocyanin at 10 °C, 16 °C, 22 °C), Leaf roll 10, 16, and 22 (presence of rolled leaves at 10 °C, 16 °C, 22 °C); and four ordinal traits: Leaf serr 10, 16, and 22 (level of leaf serration at 10 °C, 16 °C, 22 °C, five hierarchical levels ranging from 0 to 1.5); Silique 22 (silique length at 22 °C, ten hierarchical levels).

We excluded individuals with missing phenotypes, non-polymorphic SNPs, and SNPs with an MAF less than 0.10. Then, we calculated the population structure using the ADMIXTURE software (Version 1.3.0) [31], selected the best population structure matrix according to its cross-validation (CV) error (Figure S2), and inserted it into model (1), treated as a fixed-effect design matrix for each binary or ordinal quantitative trait.

The number of significant loci detected, the number of confirmed genes identified, and the computing time were used to compare the performance of each method. Bonferroni correction was used and the threshold was set to 0.05/m, where m is the number of markers involved in the real data analysis.

For each significant locus, the *Arabidopsis* Information Resource (TAIR, https://www.arabidopsis.org (accessed on 16 May 2024)) was used to mine known genes located in the vicinity of 20 kilobases (kb), and known genes have been previously confirmed to be associated with the traits of interest in the literature.

## 3. Results

### 3.1. Simulation Studies

#### 3.1.1. Statistical Power for QTN Detection

**Figure 3:**
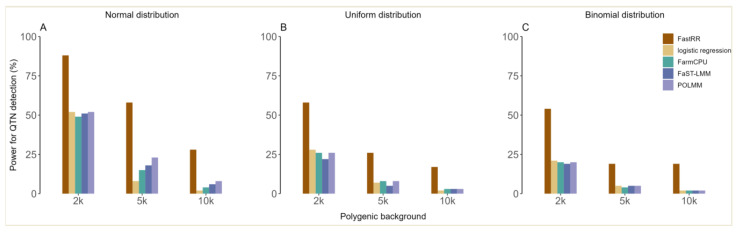
The statistical power for QTN detected by five methods in the first simulation experiment under (**A**) a normal distribution with 5 hierarchical levels, (**B**) a uniform distribution with 5 hierarchical levels, and (**C**) a binomial distribution with 2 hierarchical levels.

In the first simulation experiment, the QTN located at the 98th SNP was simulated and its heritability is detailed in Table S1, with a small effect of 0.461. For ordinal phenotypes with five hierarchical levels generated from a normal distribution, it could be seen that the statistical power of FastRR was significantly higher than four other methods (Figure 3A, Table S1). For example, under the polygenic background of 2 k, POLMM, FaST-LMM, and FarmCPU had similar power, at 52%, 51%, and 49%, respectively, but this was significantly lower than FastRR, which had 88% power. Logistic regression had the lowest power at 25% (Figure 3A, Table S1). Under the polygenic backgrounds of 5 k and 10 k, FastRR had the highest power among all five methods at 58% and 28%, respectively (Figure 3A, Table S1). With the increasing influence of the polygenic background, the statistical power of all algorithms decreased, while FastRR always performed better (Figure 3A, Table S1). As shown in Figure 3B,C, and Table S1, a similar trend was observed for a uniform distribution with five hierarchical levels and a binomial distribution with two hierarchical levels.

In the second simulation experiment, five QTNs with a heritability of 0.526~6.401% were simulated at fixed positions (Table S2). As shown in Figure S3 and Table S2, the results revealed that FastRR had the highest statistical power over four other algorithms under different polygenic backgrounds. For example, for a normal distribution, FastRR achieved power of 98%, 86%, and 52% under 2 k, 5 k, and 10 k, respectively (Figure S3A,D,G and Table S2). In contrast, FarmCPU, POLMM, and FaST-LMM had a power of 84%, 82%, and 76% under 2 k, while logistic regression achieved only 39% (Figure S3A,D,G, Table S2). The power of FastRR was similar to that of the uniform distribution and binomial distribution. For the uniform distribution (Figure S3B,E,H and Table S2), FastRR had values of 95%, 73%, and 35% under 2 k, 5 k, and 10 k, respectively; and 85%, 50%, and 28% under a binomial distribution (Figure S3C,F,I and Table S2), respectively. In particular, for the detection of small-effect loci with a heritability of less than 5% and effect values less than 1, FastRR performed with significantly higher power compared to four other methods. For instance, under the binomial distribution, the power of FastRR for the 98th marker (QTN1, *r*^2^ = 0.526~1.280%, true effect = 0.545) was at least twice that of the other methods (Figure S3C,F,I and Table S2).

In the third simulation experiment, 10 random-position QTNs of small effects were simulated with a total heritability of less than 19% and a heritability of 0.523~3.16% for each QTN. It can be seen from Figure S4 that the FastRR algorithm performed with the highest power over four other approaches for the detection of small-effect QTNs. For 2 k of ordinal simulated data, the power of FastRR exceeded 90%, followed by FarmCPU, which was slightly lower than 90%; nevertheless, the power of POLMM, FaST-LMM, and logistic regression were all below 85% (Figure S4A,B). Note that under the binomial distribution, FastRR significantly outperformed the other algorithms by more than 10% of power at 2 k and 5 k (Figure S4C). In addition, the power of all methods decreased by varying degrees as the influence of the polygenic background increased, but FastRR corrected for polygenic background was minimally affected by fluctuations in polygenic background (Figure S4) and its power was relatively stable.

#### 3.1.2. ROC Curves at Different Levels of Significance

**Figure 4:**
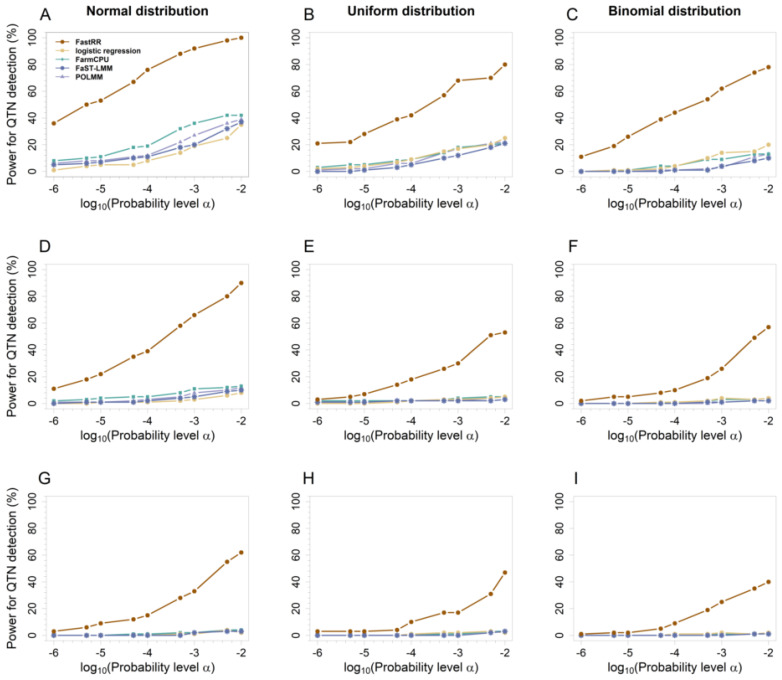
ROC curves for the five methods of the first simulation experiment. From top to bottom, each row represents 2 (**A**–**C**), 5 (**D**–**F**), and 10 (**G**–**I**) times the polygenic background, respectively.

To compare detection power across different significance thresholds, we plotted ROC curves for five methods in simulation experiments 1 and 2 (Figure 4 and Figure S5). ROC curves show that FastRR consistently outperforms other methods at various significance levels and maintains excellent detection power at low Type I error levels. In particular, FastRR demonstrated significant advantages in identifying small-effect QTN loci: in the first simulation experiment, the average statistical power of FastRR at significance levels ranging from 10^−6^ to 10^−2^ under the 2 k was found to be at least 48% greater than that of the second-best method under the normal distribution (Figure 4A), at least 38% greater under the uniform distribution (Figure 4B), and at least 42% greater under the binomial distribution (Figure 4C). In the second simulation experiment, focusing on the small-effect loci QTN1 (*r*^2^ = 0.526~1.280%, true effect = 0.545, Table S2), FastRR showed a significant advantage again, with its power being approximately 40% higher than the second-best method under all three distributions, further confirming its ability to detect small-effect QTNs (Figure S5A).

In addition, the detection ability of all methods was increasing along with the effect growth (Figure S5A–E). For example, for QTN4 (*r*^2^ = 2.105%~5.121%, true effect = 1.079, Table S2), all five methods achieved efficiencies greater than 85% when the significance level exceeded 10^−7^ under 2 k (Figure S5D). Similarly, for QTN5 (*r*^2^ = 2.631~6.401%, true effect = 1.209, Table S2), nearly all methods achieved 100% efficacy under 2 k, as evidenced by overlapping ROC curves (Figure S5E).

These results indicate that FastRR not only has excellent detection of large-effect QTNs, but also has distinct advantages in identifying small-effect QTNs.

#### 3.1.3. Accuracy for Estimated QTN Effects

The mean and MSE were used to measure the accuracy of an estimated QTN effect, and SD was used to evaluate the stability of an estimated QTN effect. We evaluated the accuracy for the fixed positions, including the first and second simulation experiments, across all five methods, as listed in Tables S1 and S2. In the first simulation experiment, POLMM and logistic regression methods exhibited the closest mean estimates to true values, followed by FastRR, FarmCPU, and FaST-LMM (Table S1). In terms of MSE, POLMM, FarmCPU, and FaST-LMM had the lowest values, followed by FastRR and logistic regression (Table S1). Regarding the stability, FastRR demonstrated SD values comparable to other methods, indicating good stability (Table S1). In the second simulation experiment, the accuracy and stability of QTN effect estimates were found to be comparable to those observed in the first simulation experiment (Table S2). These results indicate that FastRR has a robust effect estimation capability. Nevertheless, further improvements are warranted to improve the accuracy of the effect estimate when compared to the other methods.

#### 3.1.4. Computing Time

**Figure 5:**
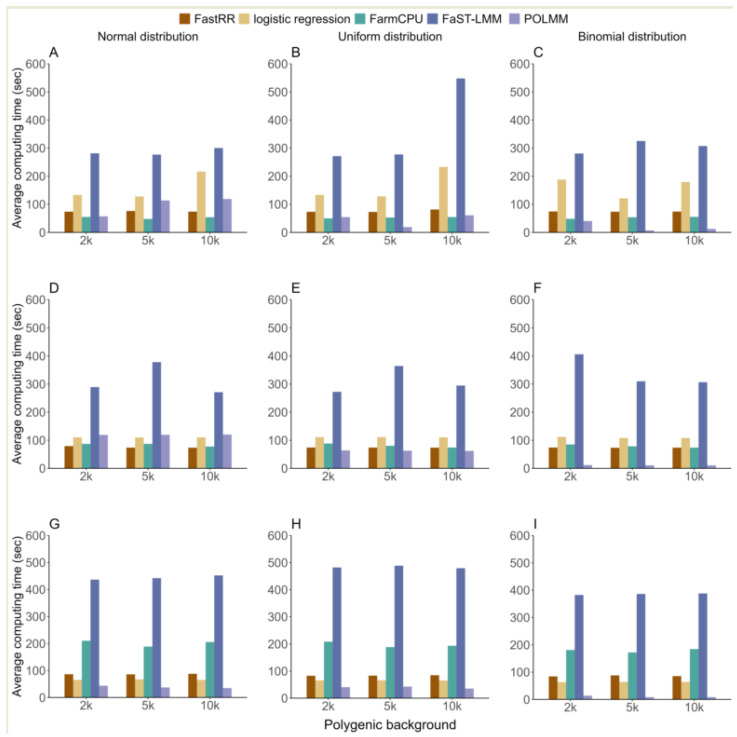
The average computing time using five methods in three simulation experiments. From top to bottom, each row represents the first (**A**–**C**), second (**D**–**F**), and third (**G**–**I**) simulation experiment, respectively.

We compared the average computing time of 100 iterations in three simulation experiments using five methods, and found that the computing time of FastRR was relatively fast and stable (Figure 5). In the first simulation experiment, FastRR was comparable in speed to POLMM and FarmCPU, all of which were finished within 75 s. Logistic regression took slightly longer than the above three methods, followed by FaST-LMM, which took 318.865 s, at least three times longer than FastRR (Figure 5A–C). The result in the second simulation experiment showed a similar pattern, with FastRR and POLMM again showing the fastest computational speeds, all completed within 75 s. FarmCPU took slightly longer, while the logistic regression and FaST-LMM methods both took over 100 s, with FaST-LMM nearly five times longer than FastRR (Figure 5D–F). In the third simulation experiment, the average computational speed of FastRR exhibited consistent performance across different polygenic backgrounds and distributions, with a time of less than 90 s, significantly lower than that of FarmCPU and FaST-LMM (Figure 5G–I).

### 3.2. Analysis of Arabidopsis Dataset

#### 3.2.1. Significant Loci Associated with Binary or Ordinal Traits

For the number of significant loci after Bonferroni correction, FastRR was significantly better than the other methods (Table 1). Specifically, for 11 out of 14 traits, including avrPphB, avrRpt2, avrB, Anthocyanin 10, 16, and 22, Leaf roll 16 and 22, Leaf serr 10 and 16, and Silique 22, it could be shown that FastRR detected more significant loci than the other methods (Table 1). FastRR identified a total of 479 significant loci associated with 14 traits, followed by FarmCPU, POLMM, and FaST-LMM with 36, 15, and 14 loci, respectively, and logistic regression did not detect any significant loci (Table 1). Notably, we found that the multi-locus approach performed better in detecting small-effect loci with low heritability compared to the single-locus approach. As shown in Table S3, the multi-locus method FastRR detected significant loci associated with known genes, with heritability ranging from 0.9% to 6.1%, and only two loci were higher than 5%; the multi-locus method FarmCPU had four loci with heritability lower than 5% and six loci with heritability higher than 5%. In contrast, the single-focus method FaST-LMM detected heritability of significant loci associated with known genes that were all above 5%. In general, FastRR demonstrates superiority in detecting small-effect loci with low heritability.

#### 3.2.2. Known Genes around Significant Loci

**Figure 6:**
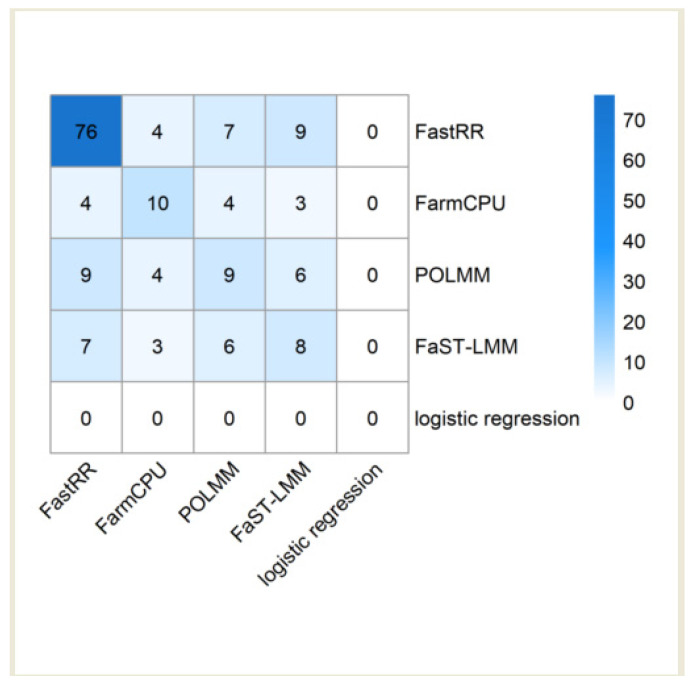
A heatmap of known genes identified by five methods for fourteen binary or ordinal traits in the *Arabidopsis* real dataset.

By retrieving the known genes on the TAIR website (https://www.arabidopsis.org/ (accessed on 16 May 2024)), FastRR detected a total of 76 known genes near the significant loci, which is 7 times more than the second ranked FarmCPU (Figure 6, Table 2 and Table S3). FarmCPU detected a total of 10 known genes; POLMM and FaST-LMM detected 9 and 8 known genes, respectively; at the same time, logistic regression did not detect any known genes.

The FastRR algorithm detected multiple gene clusters for the same trait (Table 2 and Table S3). For example, for trait avrRpm1, it was able to detect adjacent genes *AT3G59700*, *AT3G59730*, *AT3G59740*, and *AT3G59750* located at the SNP on chromosome 3 at 22,058,868 bp; for trait Leaf serr 22, it was able to detect adjacent genes *AT2G19620*, *AT2G19690*, and *AT2G19730* located at SNPs on chromosome 2 at 8,504,630 bp; for trait avrPphB, the adjacent genes *AT3G26450*, *AT3G26460,* and *AT3G26470* were detected for the SNP located at 9,700,429 bp on chromosome 3. Overall, FastRR revealed the capability to detect clusters of genes controlling target traits simultaneously.

Similarly, as shown in Figure 6, Table 2 and Table S3, known genes detected by FastRR can be simultaneously identified by other methods. For example, for trait avrPphB, genes *AT1G12210*, *AT1G12220*, and *AT1G12240*, which are adjacent to SNPs located at 4,144,558 bp and 4,150,466 bp on chromosome 1, were also detected simultaneously by FaST-LMM and POLMM. For trait avrRpt2, the gene *AT4G26120*, which is adjacent to SNPs located at 13,224,573 bp and 13,225,030 bp on chromosome 4, were also detected simultaneously by FarmCPU, POLMM, and FaST-LMM (Table 2 and Table S3). This indicates that FastRR is more reliable in mining genes.

Notably, FastRR can detect pleiotropic genes. For example, on chromosome 1, SNPs at 26,159,219 bp and 26,192,702 bp for the known gene *AT1G69588* were significantly associated with Leaf serr 22 and Leaf roll 10, respectively (Table 2 and Table S3). Known genes *AT3G59700*, *AT3G59730*, *AT3G59740*, and *AT3G59750* are located near the SNP on chromosome 3 at 22,058,868 bp and are associated with avrB and avrRpm1 (Table 2 and Table S3). Known genes *AT3G06980* and *AT3G07040* are located near the SNPs on chromosome 3 at 2,181,673 bp and 2,225,659 bp, respectively, which are also associated with avrB and avrRpm1 (Table 2 and Table S3). These findings demonstrate the ability of FastRR to detect pleiotropic genes.

#### 3.2.3. Computing Time

For all traits, FastRR was computationally much faster than all the other methods (Table 3). The average computing time of FastRR was about 60 s; logistic regression, FarmCPU, FaST-LMM, and POLMM all took at least three times as long as FastRR. Among them, logistic regression was more computationally intensive than other methods, taking more than 10 times as long as FastRR. For example, for the trait avrRpt2, FastRR took about 60 s to run, while the other four methods took more than 200 s, and logistic regression took as long as 874.381 s (Table 3). Obviously, FastRR has a significant advantage in computational speed.

## 4. Discussion

### 4.1. Advantages of FastRR over Current Methods

Most analyses of binary or ordinal traits rely on logistic or polychotomous logistic models that employ link functions to convert probabilistic models into linear models, which increases the computational expense. And methods designed for binary or continuous traits are commonly used to inappropriately analyze ordinal phenotypes, which suffer from information loss. However, FastRR offers a distinct advantage as it does not require the use of link functions; meanwhile, it could not lose useful information. This allows for direct application to binary or ordinal phenotype data. By correcting for polygenic background and population structure, and employing matrix transformation, FastRR converts these binary or ordinal traits into continuous traits instead of link functions (Equations (3)–(5)). Consequently, this approach avoids the computational complexity caused by link functions as well as the information loss caused by inappropriate methods.

For ordinal traits, we compared FastRR with two commonly used strategies: one is treating the ordinal phenotype as a continuous trait and then using FarmCPU and FaST-LMM. The other is dichotomizing the ordinal phenotype and then using logistic regression. The former violates the nature of the ordinal phenotype, and the latter could lose useful phenotypic information and statistical power [5,6,13]. We also compared FastRR with an ordinal method of POLMM. Both simulation studies of five hierarchical levels (Figure 3A,B and Figure S3A,B,D,E,G,H, and Tables S1 and S2) and real data analysis of five and ten hierarchical levels (Table 1, Table 2 and Tables S3) revealed that FastRR avoided the above strategies and employed a continuous-transformed stage in model (5), indicating the strong power to detect QTN and mine genes associated with the ordinal traits of interest.

For binary traits, we directly compared FastRR with binary methods, including logistic regression and POLMM. Meanwhile, we also compared FastRR with an inappropriate strategy of treating the binary phenotype as a continuous trait and then using FarmCPU and FaST-LMM. Through simulation studies of the binomial distribution (Figure 3C and Figure S3C,F,I, Tables S1 and S2) and real data analysis of two hierarchical levels (Table 1, Table 2 and Tables S3), it can be seen that FastRR is still reliable and valid for binary phenotype.

### 4.2. Extensive Applicability of the FastRR Method

Although the phenotype data were assumed to follow a normal distribution in model (1), the results of the simulation experiments comprehensively validated the extensive applicability of FastRR. First of all, phenotype data with varying distributions and hierarchical levels were selected for generating binary or ordinal traits, including the normal, uniform, and binomial distribution, with numbers of hierarchical level of 2 and 5. In addition, three sets of simulation studies were conducted to examine different QTNs and their heritability settings, including a single fixed-position QTN with a heritability of 0.407~1.12%, multiple fixed-position QTNs with a heritability of 0.526~6.401%, and multiple random-position QTNs with a total heritability of 7.85~18.96%. Finally, each simulation incorporated varying degrees of polygenic backgrounds with 2, 5, and 10 times the polygenic background (Figure S1, Tables S1 and S2). A total of 27 simulation scenarios were conducted. For *Arabidopsis* real data, 14 representative quantitative traits were also selected, including ten binary traits and four ordinal traits, and their hierarchical level number is 2, 5, and 10 (Figure 2, Table 1). These settings and selections fully demonstrated the wide range of applications of FastRR, demonstrating its robust detection capabilities across a variety of distributions beyond normal, including binary, multi-categorical, and other continuous distributions.

### 4.3. Prospects of the FastRR Method

FastRR has identified a series of true QTNs in simulation studies (Figure 3, Figure S3 and S4; Tables S1 and S2) and known genes in real data analysis (Figure 6, Table 2 and Tables S3). Moreover, the QTNs or genes identified by multiple methods are deemed as reliable QTNs or genes [14]. As shown in Figure 6 and Table 2 and Table S3, more known genes detected by FastRR can be simultaneously identified by other methods, indicating it is more reliable in mining genes.

As a multi-locus method that utilizes dimension reduction through variable selection, we compared FastRR with another multi-locus method, FarmCPU, andthree single-locus methods, including logistic regression, FaST-LMM, and POLMM. The results revealed that the multi-locus model considers the potential relationships between neighboring loci and explains the genetic basis of complex quantitative traits in plants better than the single-locus model, which is consistent with the previous literature [14]. The FastRR method, for example, shows excellent performance. Compared to FaST-LMM and POLMM, it increases power by at least 35% under 2 k in the first simulation experiment (Figure 3A, Table S1). Moreover, detecting small-effect loci with low heritability has been an issue in the analysis of complex categorical quantitative traits. The results reveal that for true QTN with a heritability of less than 6.401% and an effect of less than 1, FastRR demonstrates significantly superior power compared to other methods (Figure 4 and Figure S5, and Tables S1 and S2). For instance, in the second simulation experiment, the power for QTN1, which has the lowest heritability, exceeded that of other methods by at least 14% in comparison to the other four QTNs, with the advantage becoming more pronounced as the genetic background increased (Figure S3D–F, Table S2). Furthermore, in the real data, the heritability of significant loci detected by FastRR associated with known genes was consistently less than 5% in all cases (Table 2 and Table S3). In addition, FastRR, as a multi-stage algorithm, reduces the dimensionality of the raw large-scale data by variable reduction stage, and then performs the multi-locus analysis, which sharply improves the computational efficiency [22]. Regarding *Arabidopsis* real data analysis, FastRR was the fastest algorithm among all five methods (Table 3). Meanwhile, we also evaluated another three multi-locus approaches, including EBLASSO-NEG and EBLASSO-NE [23] for binary traits, and the method of Feng et al. [24] for ordinal traits. Unfortunately, none of these results have been implemented due to their low computational efficiency for large-scale data. In general, multi-locus models using variable reduction in the FastRR algorithm can be used to detect the potential relationships between neighboring loci and to mine the small-effect loci or genes with sharply rapid computation, which can be expanded to the analysis of large-scale data and multiple phenotypes. It is beneficial to allow each individual phenotype’s model to share information that can lead to better results and increased power [32]. This will be our future research.

## 5. Conclusions

In this study, the recently proposed FastRR algorithm for continuous traits was directly applied to binary and ordinal traits for genome-wide association studies. It converts the binary or ordinal trait to a continuous phenotype by polygenic background correction and special matrix transformation, instead of link functions introduced or methods inappropriately used. Compared with four other continuous/categorical data approaches, FastRR has been verified to have valid and superior performance in terms of QTN detection power, accuracy and computation in a series of simulation experiments involving different QTN settings, heritability, phenotypic distributions, hierarchical levels, and polygenic backgrounds. This superiority is particularly evident in the detection of small-effect loci, where FastRR excelled. In *Arabidopsis* real data analysis, FastRR identified 479 significant loci and 76 known genes associated with ten binary and four ordinal traits. In summary, FastRR provides an efficient GWAS tool for continuous, binary, and ordinal phenotypes.

## Figures and Tables

**Table 1:** A comparison of the number of significant loci/known genes detected by five methods for fourteen binary or ordinal traits in the *Arabidopsis* real dataset.

Trait	Hierarchical Level Number	Optimal K Value ^a^	Method				
			FastRR	Logistic Regression	FarmCPU	FaST-LMM	POLMM
avrPphB	2	3	**61/12**	0	0	7/3	6/3
avrRpm1	2	2	**71/9**	0	0	2/1	3/2
avrRpt2	2	3	**64/9**	0	8/5	3/2	2/2
avrB	2	3	**66/8**	0	8/3	2/2	4/2
Anthocyanin 10	2	5	**26/8**	0	7/1	0	0
Anthocyanin 16	2	6	**40/2**	0	0	0	0
Anthocyanin 22	2	5	**6/0**	0	1/0	0	0
Leaf roll 10	2	5	**28/4**	0	0	0	0
Leaf roll 16	2	6	**20/3**	0	0	0	0
Leaf roll 22	2	4	**18/1**	0	0	0	0
Leaf serr 10	5	4	**18/3**	0	0	0	0
Leaf serr 16	5	6	**20/9**	0	3/0	0	0
Leaf serr 22	5	4	**15/5**	0	1/0	0	0
Silique 22	10	3	**26/3**	0	8/1	0	0
total			**479/76**	0	36/10	14/8	15/9

^a^: The optimal K value corresponding to the population structure matrix for each trait. Bold represents the largest number of significant loci or known genes among the five methods.

**Table 2:** Known genes around significant loci identified by five approaches for fourteen binary or ordinal traits in the *Arabidopsis* real dataset.

Trait	Known Gene	Gene Symbol	Chr	QTN Position	Method	*p*-Value
avrPphB	*AT1G12210*	*RFL1* *RFL1*	1	4,144,558~4,150,466	1, 4, 5	3.10 × 10^−39^~1.67 × 10^−5^
	*AT1G12220*	*RPS5*				
	*AT1G12240*	*VIN2*				
	*AT3G05360*	*RLP30*	3	1,522,038	1	1.32 × 10^−4^
	*AT3G26450* *AT3G26460* *AT3G26470*		3	9,700,429	1	8.30 × 10^−5^
	*AT3G28450*	*BIR2*	3	10,662,541	1	4.50 × 10^−6^
	*AT3G26470*		3	9,603,932	1	9.64 × 10^−6^
	*AT4G08480*	*MEKK2*	4	5,412,236	1	1.31 × 10^−4^
	*AT4G23680*	*F9D16.150*	4	12,348,175	1	6.70 × 10^−5^
	*AT5G52640*	*HSP83*	5	21,355,939	1	3.52 × 10^−5^
avrRpm1	*AT1G62660*	*VI1*	1	23,220,671	1	5.93 × 10^−5^
	*AT1G32070*	*NSI*	1	11,531,340	1	1.37 × 10^−6^
	*AT2G38240*	*F16M14.17*	2	16,080,224	1	1.33 × 10^−5^
	*AT3G06980*		3	2,224,686	1, 5	1.33 × 10^−7^~1.05 × 10^−4^
	*AT3G07040*	*RPM1*	3	2,225,659~2,230,186	1, 4, 5	3.80 × 10^−9^~7.61 × 10^−6^
	*AT3G59700*	*LECRK1*	3	22,058,868	1	3.08 × 10^−4^
	*AT3G59730*	*LECRK-V.6*				
	*AT3G59740*	*LECRK-V.7*				
	*AT3G59750*	*LECRK-V.8*				
avrRpt2	*ATIG27950*	*LTPG1*	1	9,728,388	3	2.36 × 10^−8^
	*AT3G50450*	*HR1*	3	18,705,188	1	1.33 × 10^−4^
	*AT4G10490*	*DLO2*	4	6,474,413	1	2.07 × 10^−6^
	*AT4G10500*	*DLO1*	4	6,474,413	1	2.07 × 10^−6^
	*AT4G14400*	*ACD6*	4	8,276,863	1	2.89 × 10^−4^
	*AT4G12020*	*WRKY19*	4	7,216,346	3	2.58 × 10^−8^
	*AT4G12010*	*F16J13.80*	4	7,216,346	3	2.58 × 10^−8^
	*AT4G26090*	*RPS2*	4	13,224,915~13,225,030	1, 3, 4, 5	6.62 × 10^−34^~2.53 × 10^−7^
	*AT4G26120*	*F20B18.230*	4	13,224,573~13,225,030	1, 3, 4, 5	2.55 × 10^−14^~4.70 × 10^−9^
	*AT4G32551*	*RON2*	4	15,711,776	1	1.26 × 10^−5^
	*AT4G32570*	*TIFY8*				
	*AT4G35580*	*CBNAC*	4	16,896,369	1	4.46 × 10^−6^
avrB	*AT1G17250*	*RLP3*	1	5,906,765	1	1.31 × 10^−4^
	*AT1G32070*	*NSI*	1	11,531,340	1	7.55 × 10^−7^
	*AT2G46400*	*WRKY46*	2	19,033,370	3	8.62 × 10^−14^
	*AT2G46380*	*F11C10.7*	2	19,033,370	3	8.62 × 10^−14^
	*AT3G06980*		3	2,181,673~2,224,686	1, 5	2.11 × 10^−9^~2.78 × 10^−6^
	*AT3G59700*	*LECRK1*	3	22,058,868	1	2.75 × 10^−4^
	*AT3G59730*	*LECRK-V.6*				
	*AT3G59740*	*LECRK-V.7*				
	*AT3G59750*	*LECRK-V.8*				
	*AT3G07030*		3	2,227,823	4	4.08 × 10^−9^
	*AT3G07040*	*RPM1*	3	2,227,823	1, 3, 4, 5	7.21 × 10^−41^~1.58 × 10^−5^
Anthocyanin 10	*AT3G02130*	*RPK2*	3	365,429~368,145	1, 3	9.21 × 10^−8^~1.94 × 10^−4^
	*AT1G06220*	*MEE5*	1	1,921,764	1	4.78 × 10^−4^
	*AT1G06350*	*ADS4*	1	1,921,764	1	1.03 × 10^−4^
	*AT2G47700*	*RFI2*	2	19,561,188	1	1.94 × 10^−4^
	*AT3G27690*	*DEG13*	3	10,240,471	1	8.21 × 10^−5^
	*AT3G44110*	*ATJ3*	3	15,883,329	1	9.64 × 10^−5^
	*AT3G46610*		3	17,151,835	1	2.60 × 10^−4^
	*AT3G49260*	*IQD21*	3	18,257,704	1	5.11 × 10^−5^
Anthocyanin 16	*AT4G15910*	*DI21*	4	9,044,964	1	6.53 × 10^−5^
	*AT1G10120*	*CIB4*	1	3,310,433	1	8.62 × 10^−5^
Leaf roll 10	*AT1G69588*	*CLE45*	1	26,192,702	1	1.56 × 10^−4^
	*AT2G21970*	*Sep2*	2	9,349,684	1	6.91 × 10^−5^
	*AT4G38860*	*SAUR16*	4	18,146,349	1	2.66 × 10^−4^
	*AT4G39130*	*T22F8.30*	4	18,211,438	1	2.25 × 10^−4^
Leaf roll 16	*AT2G02820*	*MYB88*	2	794,422	1	3.43 × 10^−5^
	*AT1G29860*	*WRKY71*	1	10,442,076	1	1.08 × 10^−4^
	*AT2G42200*	*SPL9*	2	17,529,095	1	2.43 × 10^−5^
Leaf roll 22	*AT1G51500*	*ABCG12*	1	19,092,267	1	1.09 × 10^−4^
Leaf serr 10	*AT4G18870*	*F13C5.40*	4	10,330,949	1	4.32 × 10^−5^
	*AT4G18880*	*HSF A4A*				
	*AT5G03310*	*SAUR44*	5	809,032	1	2.32 × 10^−4^
Leaf serr 16	*AT1G29420*	*SAUR61*	1	10,298,618	1	1.67 × 10^−4^
	*AT1G29430*	*SAUR62*				
	*AT1G29460*	*SAUR65*				
	*AT1G29640*	*F15D2.20*	1	10,301,221	1	1.50 × 10^−4^
	*AT1G51760*	*IAR3*	1	19,198,124	1	5.76 × 10^−5^
	*AT1G51780*	*ILL5*				
	*AT2G01420*	*PIN4*	2	180,480	1	7.40 × 10^−5^
	*AT4G11880*	*AGL14*	4	7,137,798	1	1.83 × 10^−4^
	*AT4G16150*	*CAMTA5*	4	9,154,429	1	2.64 × 10^−4^
Leaf serr 22	*AT1G14350*	*FLP*	1	4,923,296	1	1.82 × 10^−4^
	*AT1G69588*	*CLE45*	1	26,159,219	1	2.46 × 10^−4^
	*AT2G19620*	*NDL3*	2	8,504,630	1	1.60 × 10^−4^
	*AT2G19690*	*F6F22.28*				
	*AT2G19730*	*EL28Z*				
Silique 22	*AT4G36020*	*CSP1*	4	17,057,521	1	7.87 × 10^−5^
	*AT1G65500*	*F5I14.4*	1	24,373,119	1	6.57 × 10^−4^
	*AT1G77080*	*MAF1*	1	28,946,359	3 3	4.08 × 10^−8^
	*AT3G02130*	*RPK2*	3	399,288	1	4.01 × 10^−4^

Note: Only the results of 13 traits, except for Anthocyanin 22, were listed in the table because no known genes were found for Anthocyanin 22. GWAS methods are indicated as 1 (FastRR), 2 (logistic regression), 3 (FarmCPU), 4 (FaST-LMM), and 5 (POLMM).

**Table 3:** A comparison of computation times (in seconds) for five methods applied to fourteen binary and ordinal traits in the *Arabidopsis* real dataset.

Trait	Hierarchical Level Number	Method				
		FastRR	Logistic Regression	FarmCPU	FaST-LMM	POLMM
avrPphB	2	**56.694**	820.554	580.096	184.906	639.752
avrRpm1	2	**60.543**	777.928	569.730	193.724	577.811
avrRpt2	2	**60.361**	874.381	382.826	207.916	661.600
avrB	2	**61.786**	819.524	308.278	276.553	571.332
Anthocyanin 10	2	**63.490**	966.688	170.400	337.322	602.908
Anthocyanin 16	2	**63.124**	968.497	170.031	345.887	562.880
Anthocyanin 22	2	**63.483**	966.562	187.875	353.152	712.398
Leaf roll 10	2	**63.700**	981.194	190.665	357.159	594.057
Leaf roll 16	2	**64.672**	981.164	184.358	368.970	534.023
Leaf roll 22	2	**63.868**	962.953	187.759	397.664	584.306
Leaf serr 10	5	**64.083**	976.726	176.467	359.268	781.890
Leaf serr 16	5	**63.196**	959.743	175.202	373.683	917.177
Leaf serr 22	5	**66.011**	992.060	177.251	352.197	569.712
Silique 22	10	**62.799**	786.014	376.251	304.925	603.166

Note: Bold represents the fastest computing time among the five methods.

## Data Availability

Data recorded in the current study are available at https://www.arabidopsis.org/ (accessed on 16 May 2024). Further inquiries can be directed to the corresponding author.

## Supplementary Materials

The following supporting information can be downloaded at https://www.mdpi.com/article/10.3390/plants13172520/s1, Figure S1: various phenotypic distributions and hierarchical levels in three simulation experiments. From left to right, each column illustrates a different distribution. From top to bottom, each row represents the first (A–C), second (D–F), and third (G–I) simulation experiment, respectively. Figure S2: the relationship between CV error and K values for group structure of 14 binary or ordinal traits in the *Arabidopsis* real dataset. Figure S3: statistical power for 5 fixed-position QTNs detected by five methods in the second simulation experiment. From left to right, each column illustrates a different distribution. From top to bottom, each row represents 2 (A–C), 5 (D–F), and 10 (G–I) times the polygenic background, respectively. Figure S4: statistical power for 10 random-position QTNs detected by five methods in the third simulation experiment under (A) normal distribution with 5 hierarchical levels, (B) uniform distribution with 5 hierarchical levels, and (C) binomial distribution with 2 hierarchical levels. Figure S5: ROC curves for five fixed-position QTNs (A:QTN1; B:QTN2; C:QTN3; D:QTN4; E:QTN5) for five methods in the second simulation experiment. Each figure is given from left to right; each column illustrates a different distribution. From top to bottom, each row represents 2, 5, and 10 times the polygenic background, respectively. Table S1: statistical power, and accuracy in the detection of QTN using five methods in the first simulation experiment. Table S2: statistical power, and accuracy in the detection of QTN using five methods in the second simulation experiment. Table S3: significant QTNs and their known genes detected using five methods for 14 binary or ordinal traits in the *Arabidopsis* real dataset.

## Abbreviations

Bayes-GLMM, generalized linear mixed model in a Bayesian framework; BOLT-LMM, fast Bayesian mixed-model association; DRR, deshrinking ridge regression; EBLASSO-NEG, empirical Bayesian LASSO with normal, exponential, and gamma hierarchical prior; EBLASSO-NE, empirical Bayesian LASSO with normal and exponential hierarchical priors; EMMA, efficient mixed-model association; FastRR, fast multi-locus ridge regression; FaST-LMM, factored spectrally transformed linear mixed model; FarmCPU, fixed and random model circulating probability unification; FASTmrEMMA, fast multi-locus random-SNP-effect EMMA; FASTmrMLM, fast mrMLM; FastGWA, MLM-based GWA analysis; FEM, fixed-effect model; GEMMA, genome-wide efficient mixed linear model association; GLM, generalized linear model; GLMM, generalized linear mixed model; GMMAT, generalized mixed-model association test; GWAS, genome-wide association studies; LASSO, least absolute shrinkage and selection operator; MAF, minor allele frequency; MLM, mixed linear model; mrMLM, multi-locus random-SNP-effect MLM; MSE, mean squared error; pLARmEB, polygenic-background-control-based least angle regression plus empirical Bayes; POLMM, proportional odds logistic mixed model; QTN, quantitative trait nucleotide; REM, random effect model; ROC, receiver operating characteristic; SAIGE, scalable and accurate implementation of generalized mixed model; SNP, single-nucleotide polymorphism; TAIR, the *Arabidopsis* Information Resource.
