# Supplementary figures and images for: An Extended Application of the Fast Multi-Locus Ridge Regression Algorithm in Genome-Wide Association Studies of Categorical Phenotypes
Source: Plants (Basel). 2024 Sep 7;13(17):2520. doi: 10.3390/plants13172520 (PMC11397509; doi:10.3390/plants13172520)

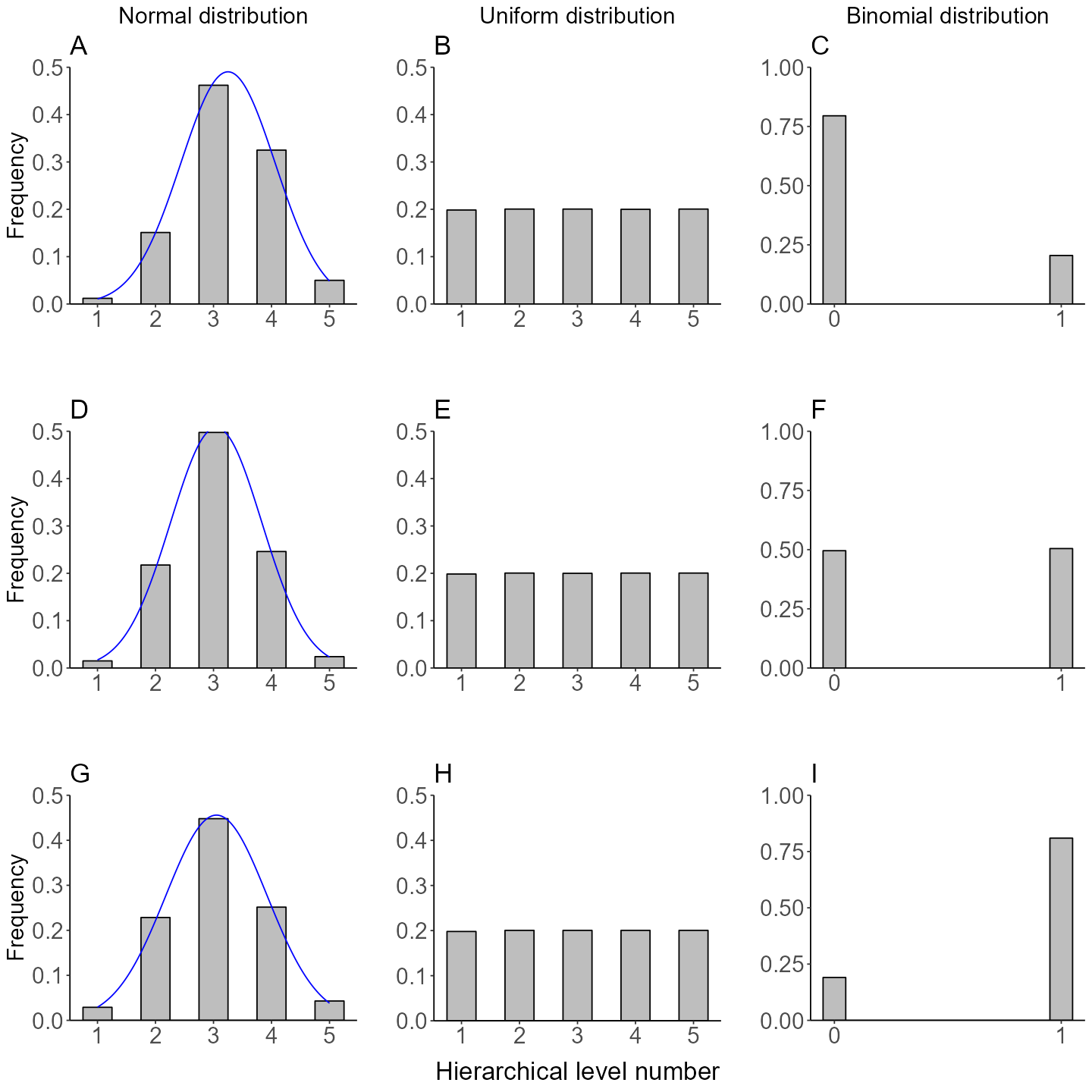

Supplement: Supplementary file 1 [file plants-13-02520-s001.zip › plants-3152741-supplementary_2/Figure S1.tif]

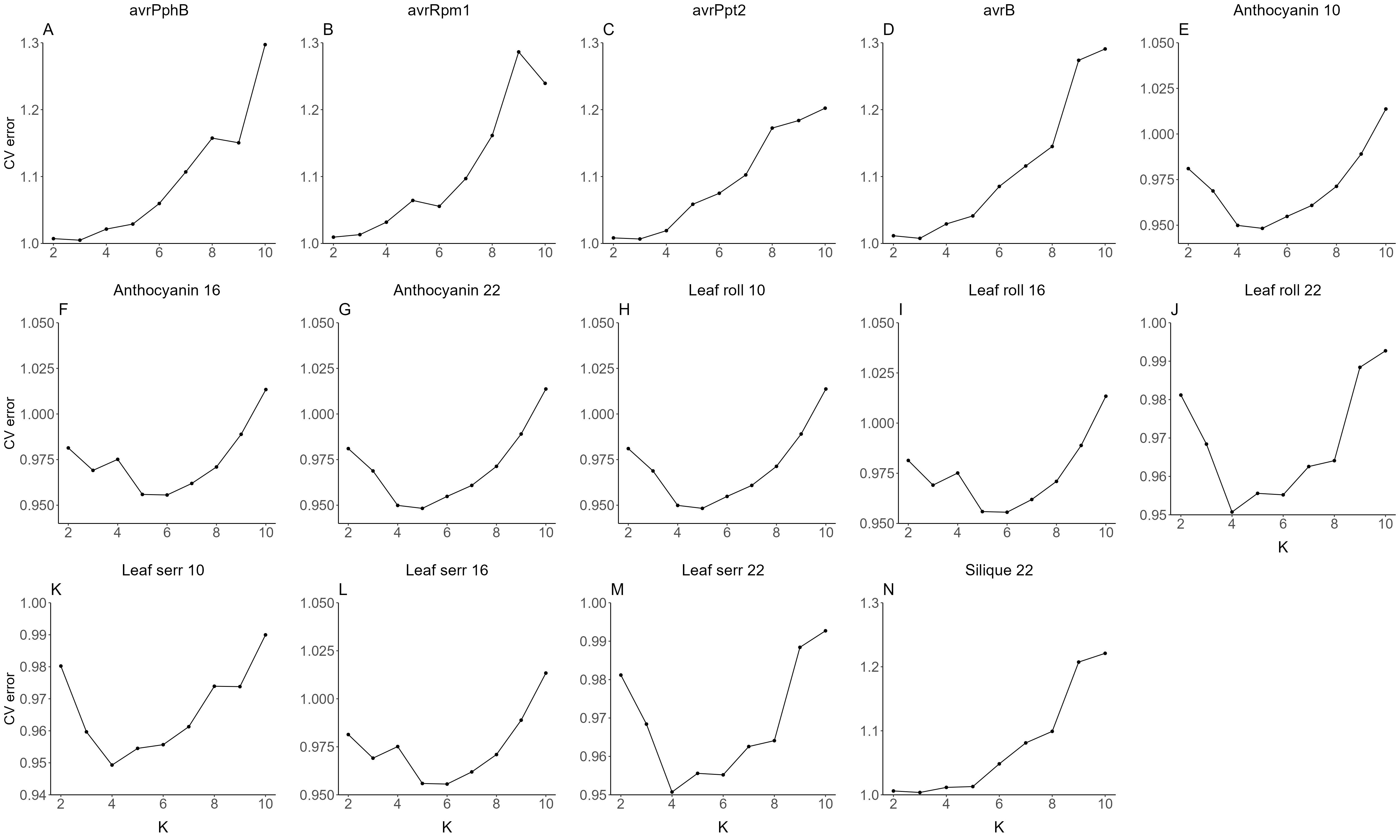

Supplement: Supplementary file 1 [file plants-13-02520-s001.zip › plants-3152741-supplementary_2/Figure S2.tif]

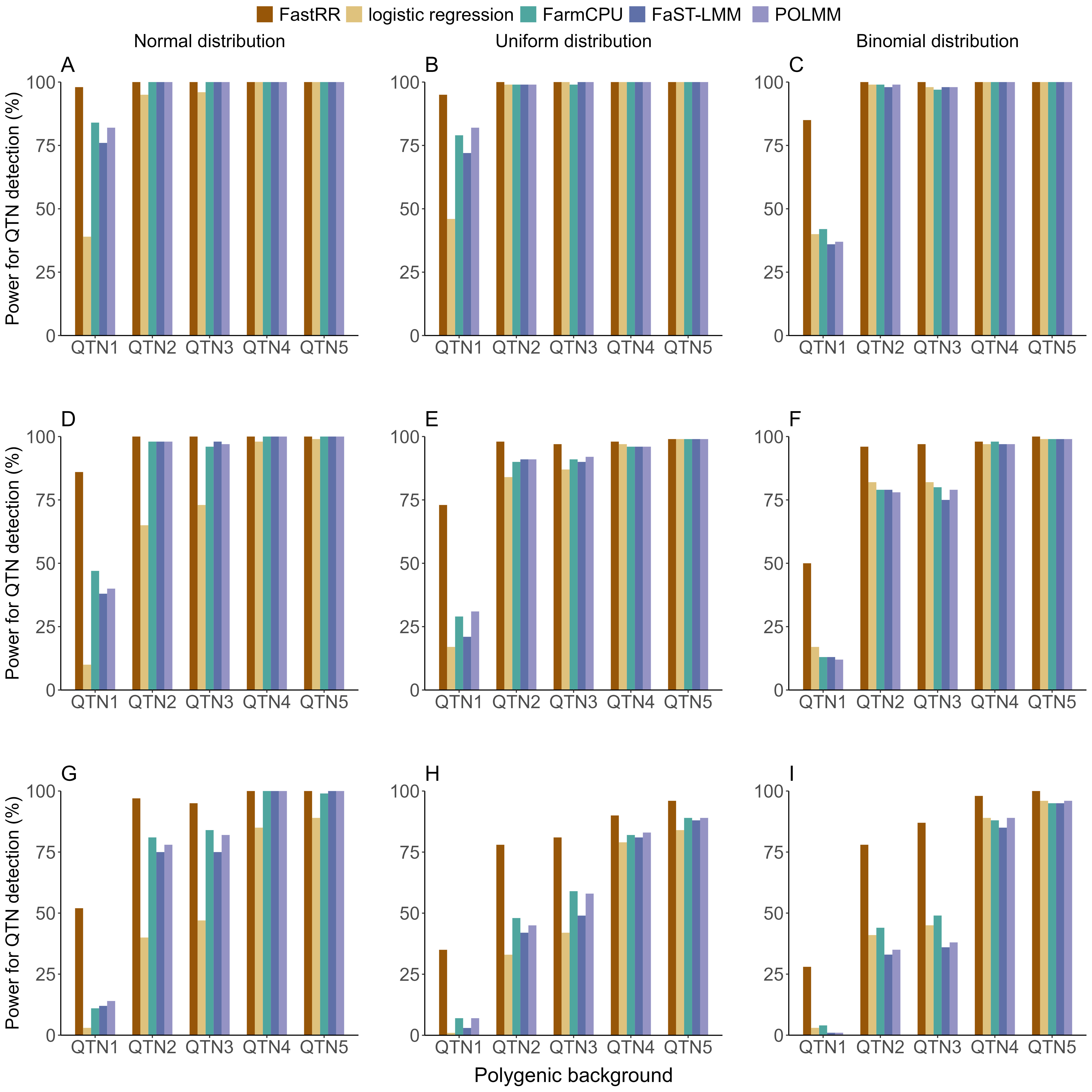

Supplement: Supplementary file 1 [file plants-13-02520-s001.zip › plants-3152741-supplementary_2/Figure S3.tif]

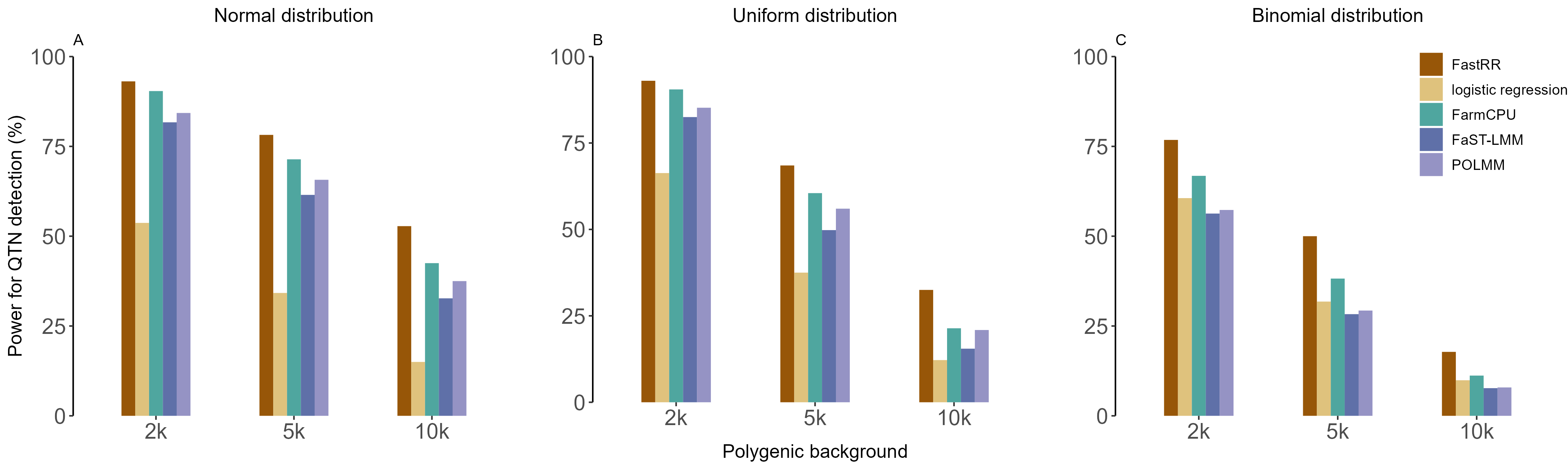

Supplement: Supplementary file 1 [file plants-13-02520-s001.zip › plants-3152741-supplementary_2/Figure S4.tif]

A

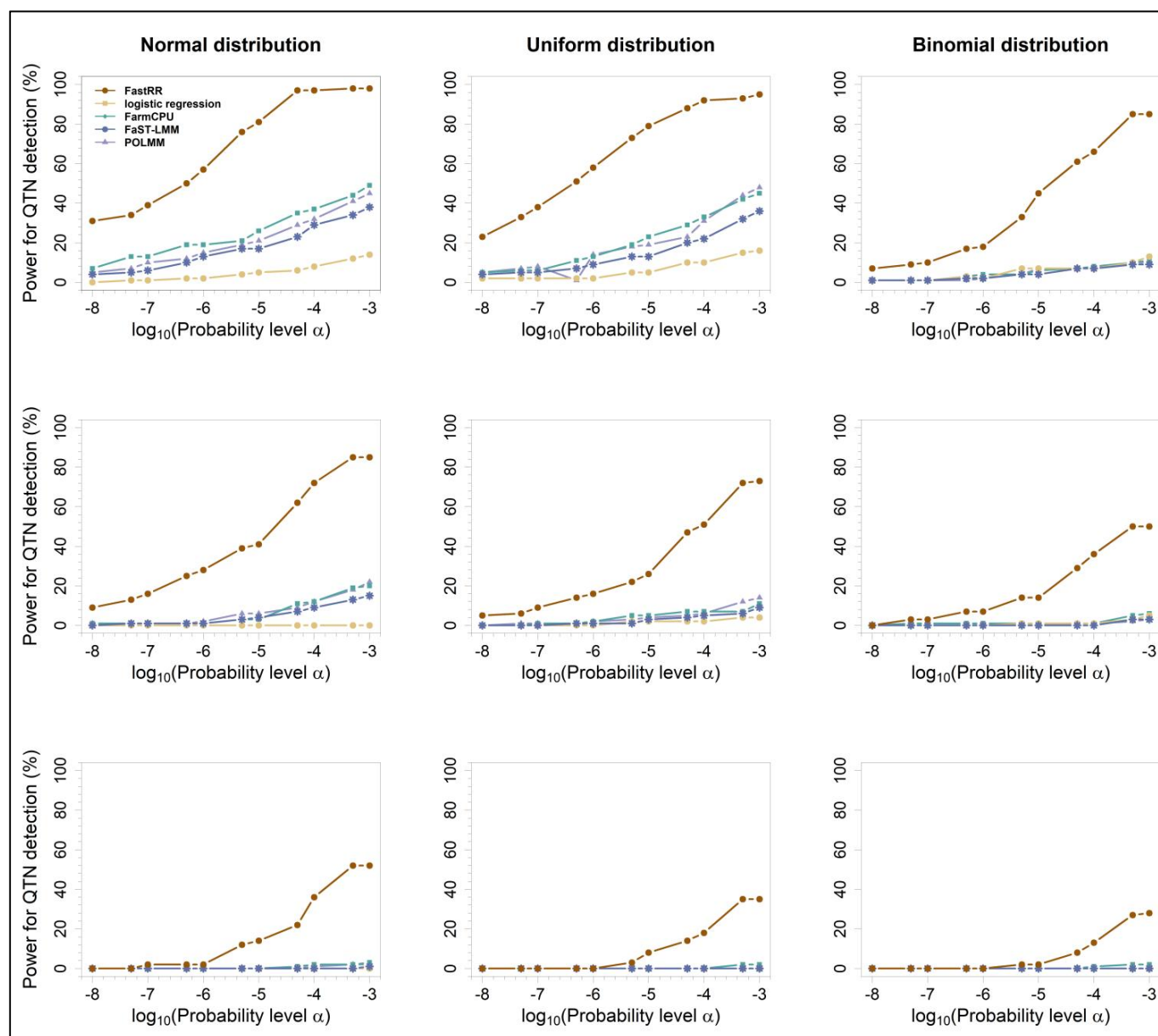

B

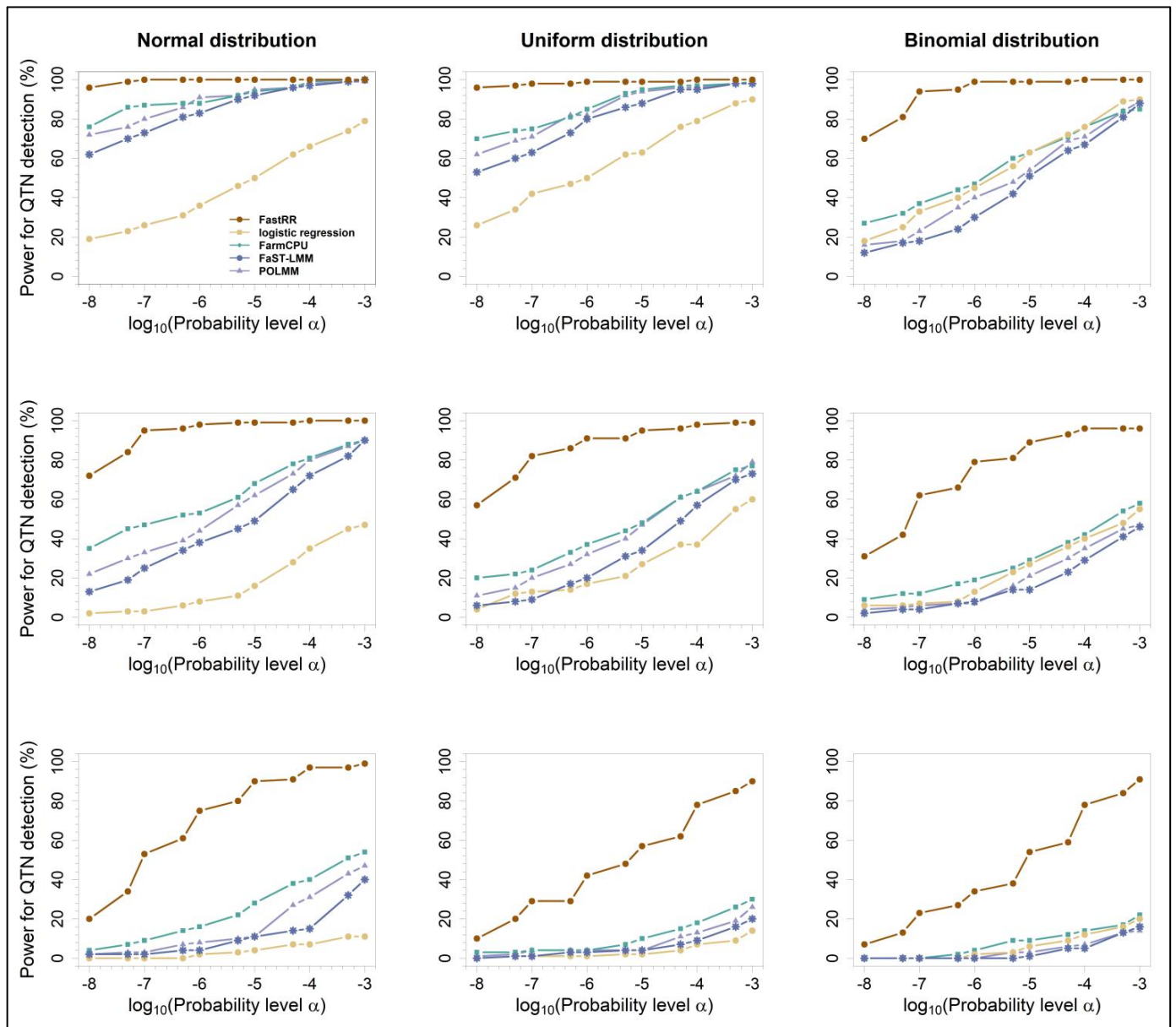

C

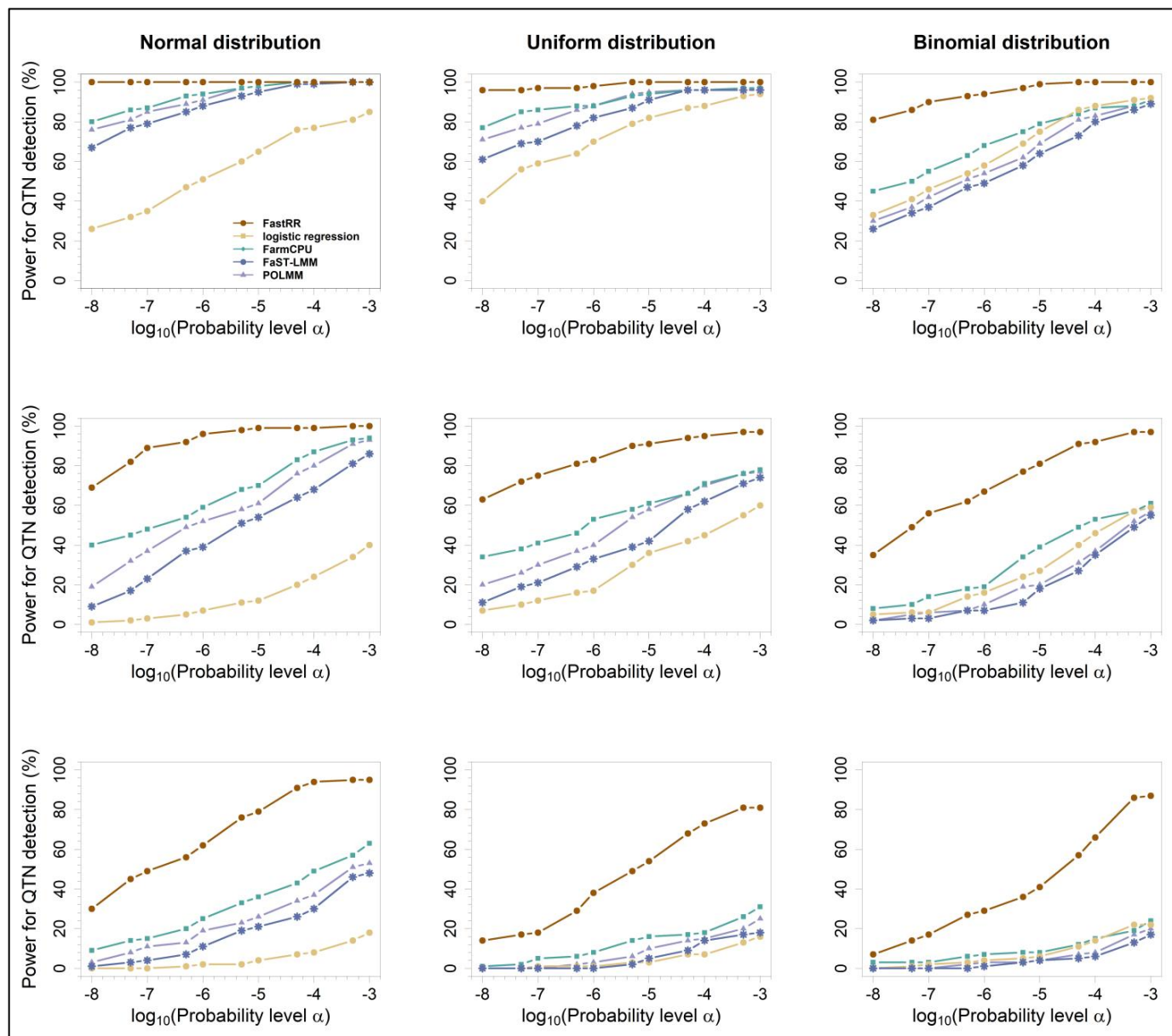

D

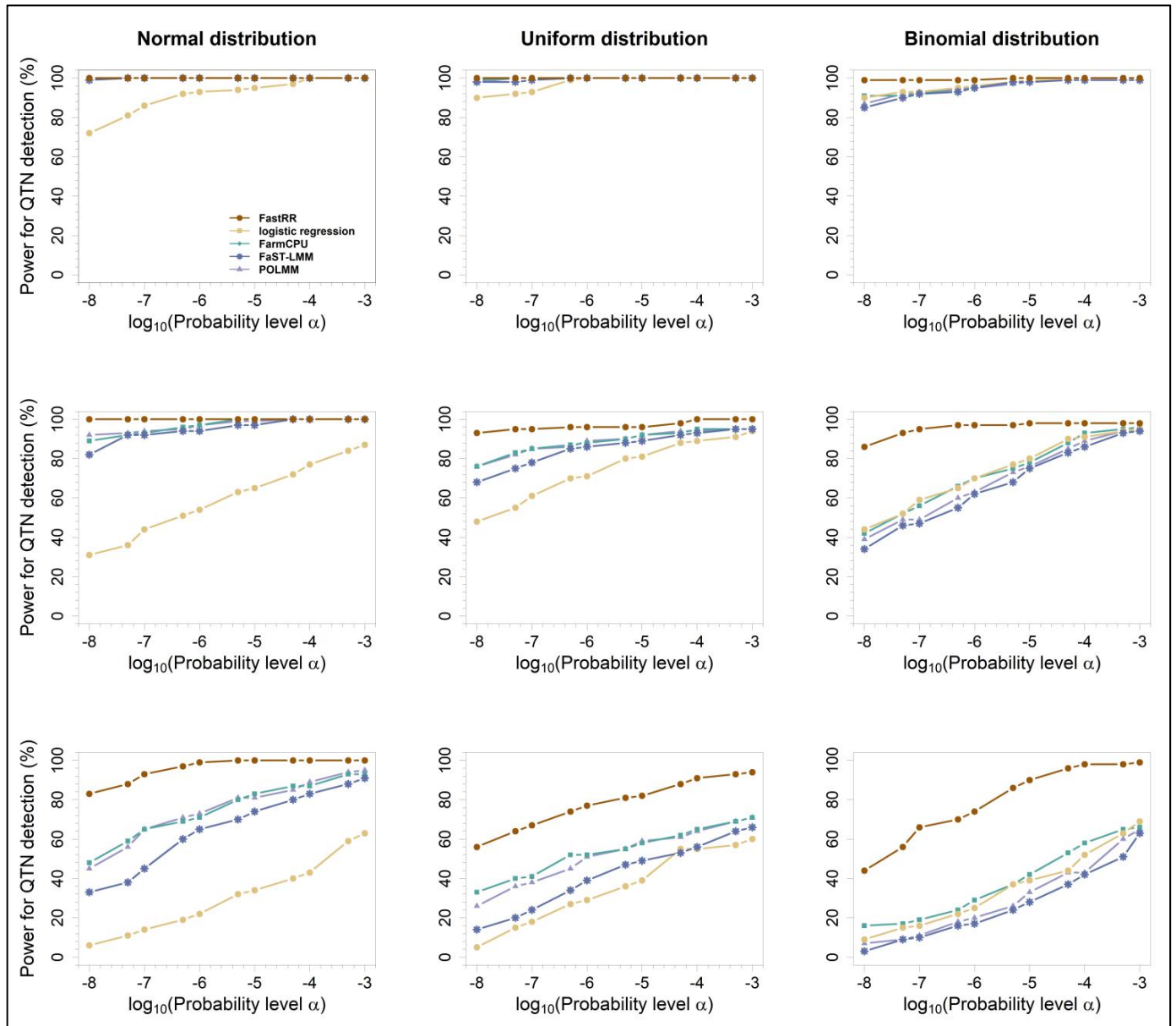

E

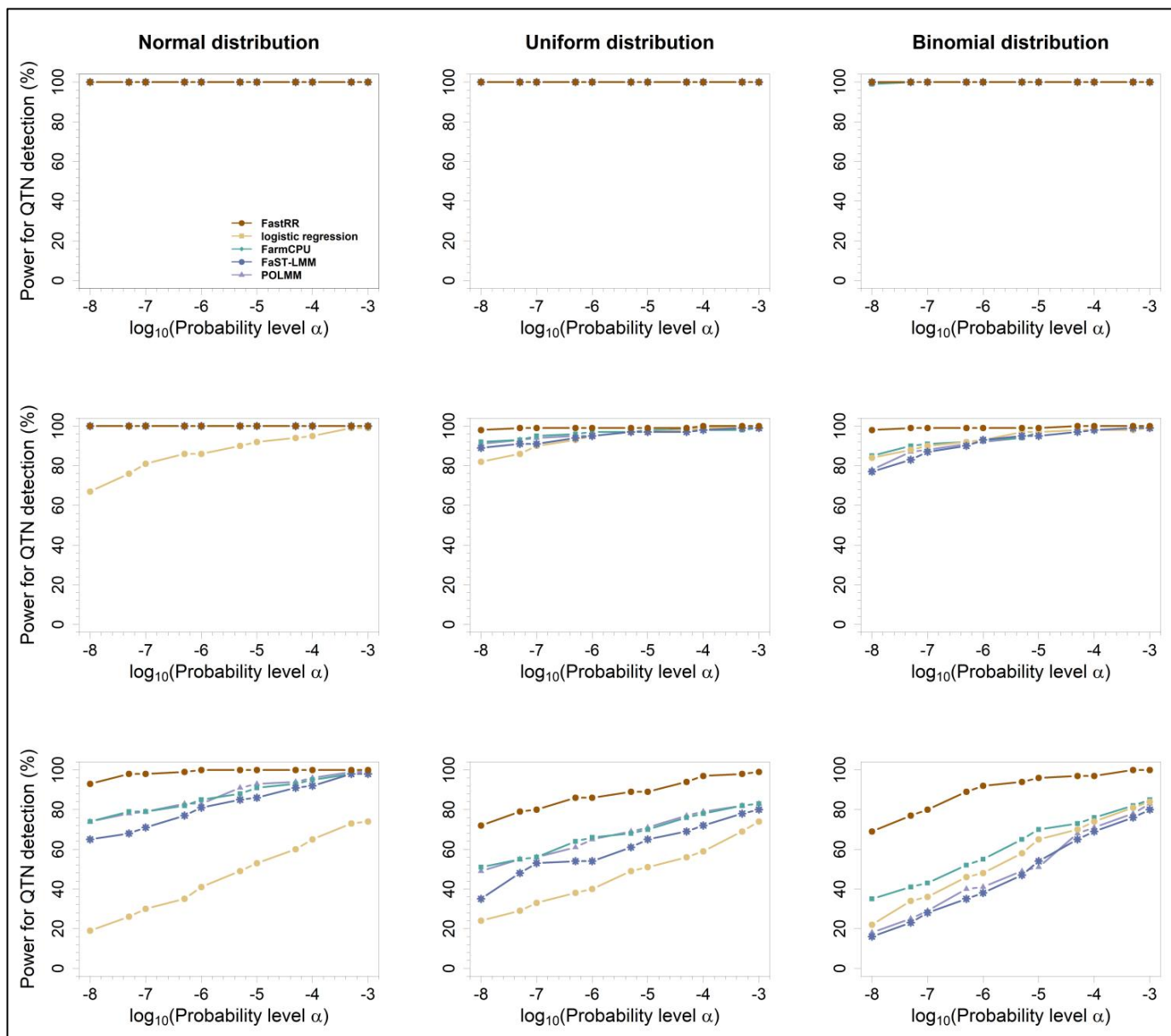

Supplement: Supplementary file 1 [file plants-13-02520-s001.zip › plants-3152741-supplementary_2/Figure S5.pdf]
